# Data for transcriptomic and iTRAQ proteomic analysis of *Anguilla* japonica gills in response to osmotic stress

**DOI:** 10.1016/j.dib.2015.02.012

**Published:** 2015-03-04

**Authors:** William Ka Fai Tse, Jin Sun, Huoming Zhang, Keng Po Lai, Jie Gu, Alice Yu Sheung Law, Bonnie Ho Yee Yeung, Sheung Ching Chow, Jian-Wen Qiu, Chris Kong Chu Wong

**Affiliations:** a Department of Biology, Hong Kong Baptist University, Hong Kong SAR, China; b School of Biological Science, the University of Hong Kong, Hong Kong SAR, China; c Croucher Institute for Environmental Sciences, Hong Kong Baptist University, Hong Kong SAR, China

## Abstract

This article contains data related to the two research articles titled Transcriptomic and iTRAQ proteomic approaches reveal novel short-term hyperosmotic stress responsive proteins in the gill of the Japanese eel (*Anguilla japonica*) (Tse et al. [1]) and iTRAQ-based quantitative proteomic analysis reveals acute hypo-osmotic responsive proteins in the gills of the Japanese eel (*Anguilla japonica*) (Tse et al. [2]). The two research articles show the usefulness of combining transcriptomic and proteomic approaches to provide molecular insights of osmoregulation mechanism in a non-model organism, the Japanese eel. The information presented here combines the raw data from the two studies and provides an overview on the physiological functions of fish gills.

Specifications table

**Table:** 

Subject area	Biology

More specific subject area	Eel transcriptome and proteomics
Type of data	Table, figure
How data was acquired	Roche 454 sequencer, iTRAQ mass spectroscopy, instrument including Thermo Electron LTQ-Orbitrap Velos coupled with a Proxeon Easy-nLC
Data format	Analyzed
Experimental factors	**Transcriptomic:** PolyA-RNA was purified using the Poly(A)Purist™ MAG Kit and fragmented using the RNA fragmentation buffer. Double stranded cDNA was synthesized using the cDNA Synthesis System Kit with randomprimers and purified using AMPure beads.**Proteomics:** Protein was chemically reduced with 5 mM tris carboxyethyl phosphine hydrochloride for 60 min at 37 °C and alkylated with 10 mM methylethanethiosulfonate (MMTS) for 20 min at room temperature. Samples were then diluted 7-fold with 50 mM triethylammonium bicarbonate prior to digestion by sequencing grade trypsin for 16 h at 37^o^C in a 1:50 trypsin-to-protein mass-ratio. The digests were desalted using Sep-Pak C18 cartridges and dried in SpeedVac.
Experimental features	11,033 contigs and 965 proteins were identified from the RNA-seq and iTRAQ respectively Functional analysis such as GO, KEGG, and PPI network were performed.
Data source location	Hong Kong
Data accessibility	*The data are with this article and related to Tse* et al. *2013, 2014*[1,2]

Value of the data [describe in 3–5 bulleted points why this data is of value to the scientific community]

• The data provides addition information and analysis that based on Tse et al. [1,2].
• The data provides an overview of eel gill biological functions.
• The data further supported the use of transcriptomic and proteomics technologies in the non-model organism.

## 1 Data

The data are directly available in this article and related to our previous publications Tse et al. [1] and Tse et al. [2].

**Fig. 1:**
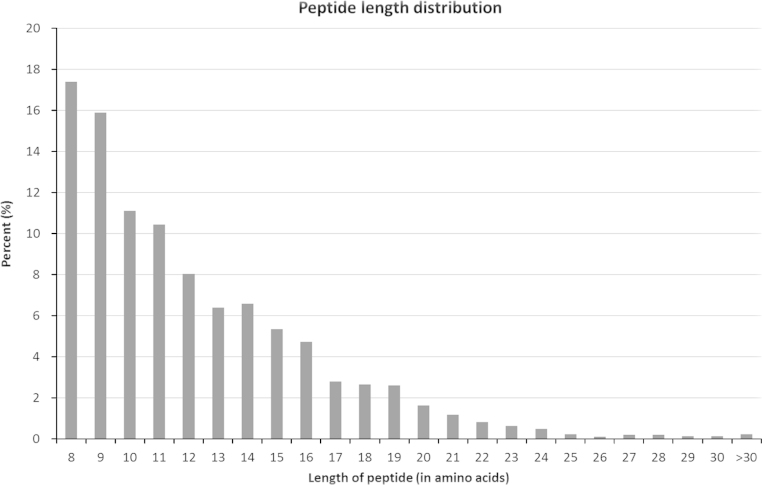
The distribution of length and number of peptides from iTRAQ proteomics.

**Fig. 2:**
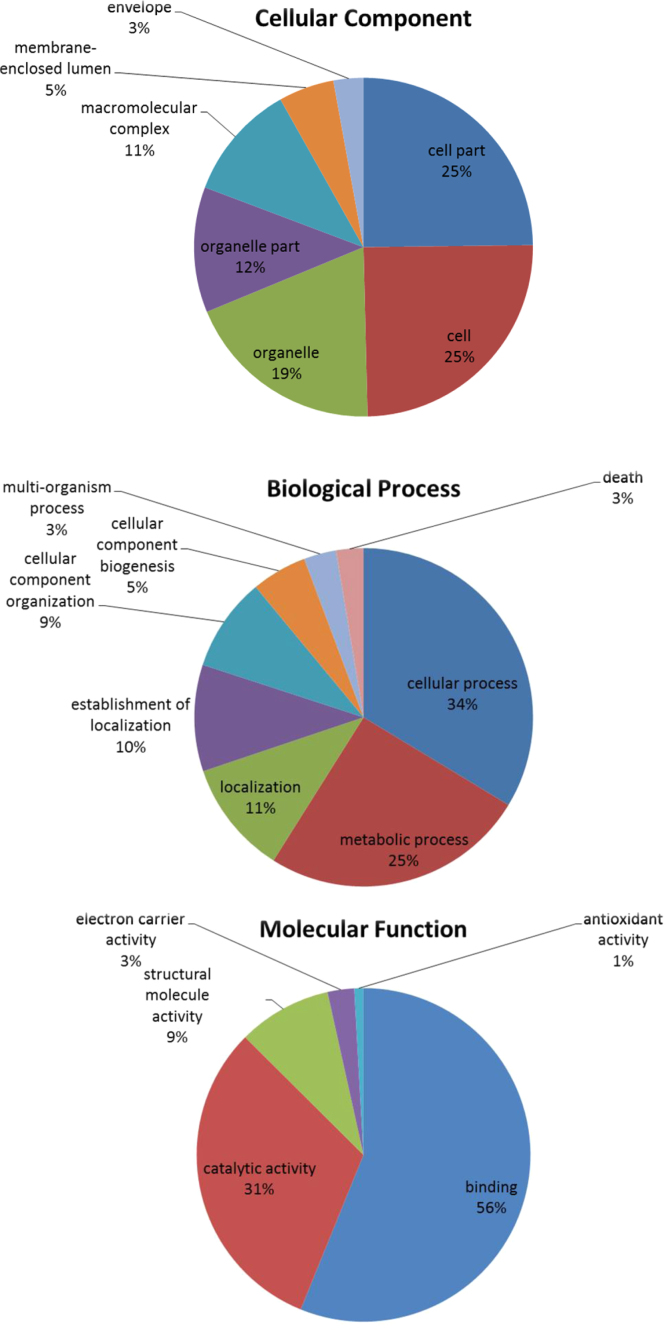
The Gene ontology (GO) assignment of eel gill proteins.

Eels are euryhaline fish that migrates between fresh water (FW) and seawater (SW) in its life cycle. In order to identify the acute osmo-responsive proteins in the fish gills, we have generated the first transcriptome profile of eel gills in 2013, and used the data for our iTRAQ proteomics analysis [1,2]. We extracted the gill total RNA from eels reared in both freshwater (FW) and seawater (SW) to generate a pooled sample for RNA-seq, which resulted in 11,033 contigs. The detailed annotation information is tabulated in the Supplementary file 1. The sequences are deposited in the NCBI Sequence Read Archive with the accession number SRX247092, and the assembled contigs are deposited in the TSA (GAGT00000000). For iTRAQ proteomics experiments, eel gill protein samples in four different treatments were extracted and labeled, which were FW (114); SW (115), FW to 6 h SW (116), and SW to 6 h FW (117). A total of 965 proteins were identified [1,2]. The length distribution and numbers of peptides are shown in Fig. 1. In order to give an overview of the gill functions, using The Database for Annotation, Visualization and Integrated Discovery (DAVID), we analyzed all of the identified proteins, regardless their responses to different osmotic stresses. In the Gene Ontology (GO) analysis, we found that cellular process, cell and cell part, and binding were the highest enriched terms in the categories of Biological Process, Cellular Component, and Molecular Function, respectively (Fig. 2). While in the Kyoto Encyclopaedia of Genes and Genomes (KEGG) analysis, 34 pathways were enriched (Supplementary file 2). Among these, two osmoregulatory related terms (tight function and adherens junction) were found. In addition, numerous metabolic pathways such as glycolysis/gluconeogenesis, citrate cycle, and fatty acid metabolism were enriched in the KEGG analysis. The data supported that the fish gill has a great demand for energy metabolisms to support its multiple functions [3]. Lastly, the overview of eel gill PPI network was shown for the first time (Supplementary file 3).

## 2 Experimental design, materials and methods

### 2.1 Animals

Japanese eels weighing 500–600 g were bought from a local fish market. The fish were kept in two 40-L glass tanks with charcoal-filtered aerated FW or SW, and were maintained at 18–20 °C under a 12-h light:12-h dark photoperiod for at least 3 weeks. Ten-liter of the water was changed every 3 days. For experiments to induce osmotic stress, eels were transferred directly from FW to SW tanks or SW to FW tanks. At 6 h of the post transfer, the fish were euthanized in 0.1% MS222 solution. Gill tissues were perfused with a buffered saline (130 mM NaCl, 2.5 mM KCl, 5 mM NaHCO_3_, 2.5 mM glucose, 2 mM EDTA, and 10 mM HEPES, pH 7.7) to remove blood cells. The gill arches were excised, washed and were then collected for RNA and protein extraction. Fully adapted FW or SW fish were sampled in the same way. All experimental procedures were approved by the Hong Kong Baptist University, Hong Kong Special Administrative Region.

### 2.2 RNA extraction, cDNA library construction, and sequencing

Total RNA was extracted from gills of freshwater (FW) and seawater (SW) adapted fish using TRIzol reagent (Invitrogen). RNA samples were pooled from gills of FW and SW eels. PolyA-RNA was purified using the Poly(A)Purist^TM^ MAG kit (Ambion) and cut by the RNA fragmentation solution. Double stranded cDNA was synthesized using the cDNA Synthesis System Kit (Roche) with random primers and was purified using AMPure beads (Agencourt). DNA sequencing was performed following protocols for the Genome Sequencer GS FLX Titanium System (Roche Diagnostic). Sequencing reads were then trimmed to eliminate low quality sequences and were assembled by GS De Novo Assembler. The raw sequencing data are deposited in the NCBI Sequence Read Archive with the accession number SRX247092, and the assembled contigs are deposited in the TSA (GAGT00000000) [1].

### 2.3 Protein extraction, iTRAQ labeling and mass spectrometry (MS)

Gill tissues were homogenized in a lysis buffer containing 8 M urea and 40 mM HEPES, pH 7.4. Sonication was used to shear the cells, and cell debris was removed by centrifugation at 15,000*g* for 10 min. A 2-D Clean-up Kit (Bio-Rad) was used to purify the protein, and protein concentrations were determined using the RC-DC Protein Assay Kit (Bio-Rad). 200 µg of protein from each sample was chemically reduced with 5 mM tris carboxyethyl phosphine hydrochloride for 60 min at 37 °C and alkylated with 10 mM methylethanethiosulfonate (MMTS) for 20 min at room temperature. Samples were then diluted 7-fold with 50 mM triethylammonium bicarbonate prior to the digestion by sequencing grade trypsin (Promega) for 16 h at 37 °C in a 1:50 trypsin-to-protein mass-ratio. The digests were desalted using Sep-Pak C18 cartridges (Waters) and dried in a SpeedVac (Eppendorf). Peptides from the FW, SW, 6 h post FW-to-SW transfer, and 6 h post SW-to-FW transfer fish gill samples were labeled with the 114, 115, 116, and 117 iTRAQ reporters, respectively. Peptide fractionation was performed using a strong cation exchange column as described [4].

Each of the fractions was analyzed three times using an LTQ-Orbitrap Velos (Thermo Electron) coupled with an Easy-nLC (Proxeon). For each analysis, a peptide was separated in a C18 capillary column (Michrom Bioresouces) with a 90- min gradient composed of 5 min of 100% A (0.1% formic acid in water), 50 min of 0–30% B (0.1% formic acid in acetonitrile), 15 min of 30–80% B, maintenance at 80% B for 10 min, and re-equilibration at 100% A for 10 min. A full mass spectrometry (MS) scan (350–1600 m/z range) was acquired in the Orbitrap at a resolution of 30,000 (at 400 m/z); the maximum ion accumulation time and target value were maintained as 2 s and 1e^6^. Precursor ion charge state screening was activated. The three most abundant ions with multiple charges and above the 500-count threshold were selected for fragmentation by high-energy collision-induced dissociation (HCD) in C-trap and collision-induced dissociation (CID) in linear trap quadrupole with an isolation width of 3.0 (m/z). Dynamic exclusion was activated for this process with a repeat count of 2, exclusion duration of 45 s, and ±5 ppm mass tolerance. The parameters in HCD fragmentation were: full scan with Fourier transform MS at resolution of 7500 (at 400 m/z) in a centroid mode, a target value of 5e^4^, normalized collision energy (NCE) of 50%, and an activation time of 10 ms. For CID, the NCE, activation Q, and activation time were set at 35%, 0.25, and 10 ms, respectively.

### 2.4 Protein identification and quantification

The MASCOT generic files (*mgf*) were gained in Proteome Discoverer 1.0 (Thermo Fisher Scientific) for both HCD and CID data. The mass and intensity information of 114, 115, 116, and 117 iTRAQ ions were also extracted from HCD *mgf* files and replaced the corresponding spectra in CID *mgf* files using a python script. All the eel protein sequences including 46,968 unique sequences were reversed and concatenated with the forward versions in order to fulfill the requirements of the “target-decoy” database search strategy to determine the false discovery rate (FDR). The normalized *mgf* files of three replicates were submitted through Mascot (version 2.3.02) to search the protein database. The following settings were used in database search: tolerance for peptides and fragments in ion trap: 8 ppm and 0.6 Da, respectively; fixed modification: MMTS (cysteine); variable modification: oxidation (methionine) and two tolerant missed cleavages. Peptides that contained 7 or fewer amino acids were removed from the protein identification and quantification. To maintain an FDR of less than 1% at both the peptide and protein levels, cut-off scores were dynamically assigned to each dataset. Proteins containing at least two spectra were used for quantification [1,2].

### 2.5 Bioinformatics

GO and KEGG pathway analysis of the identified 965 proteins was conducted by DAVID [5]. A protein–protein interaction (PPI) spider was used to construct an overview of the PPI information based on the global PPI network from the IntAct database [6]. The *Homo sapiens* orthologs were based on homolog match using all of the detected eel proteins employed in the PPI spider. We used the gene symbols of each protein in the PPI spider submission gene list. The D2 model, which corresponded to 1 maximal intermediate protein allowed between two input proteins, was considered. The enrichment significance was further assessed by a *p*-value probability, and the generated “.xgmml” file was visualized by Cytoscape v 3.2.0.

## Conflict of interest

None declared.

## Supplementary materials

S1: The detailed annotation information of eel gills from RNA-seq

S2: Enriched KEGG pathways in eel gills

S3: PPI network of eel gills

## Footnotes

Appendix A Supplementary data associated with this article can be found in the online version at doi:10.1016/j.dib.2015.02.012.
