# Supplementary figures and images for: Data for transcriptomic and iTRAQ proteomic analysis of Anguilla japonica gills in response to osmotic stress
Source: Data Brief. 2015 Mar 4;3:120–5. doi: 10.1016/j.dib.2015.02.012 (PMC4510098; doi:10.1016/j.dib.2015.02.012)

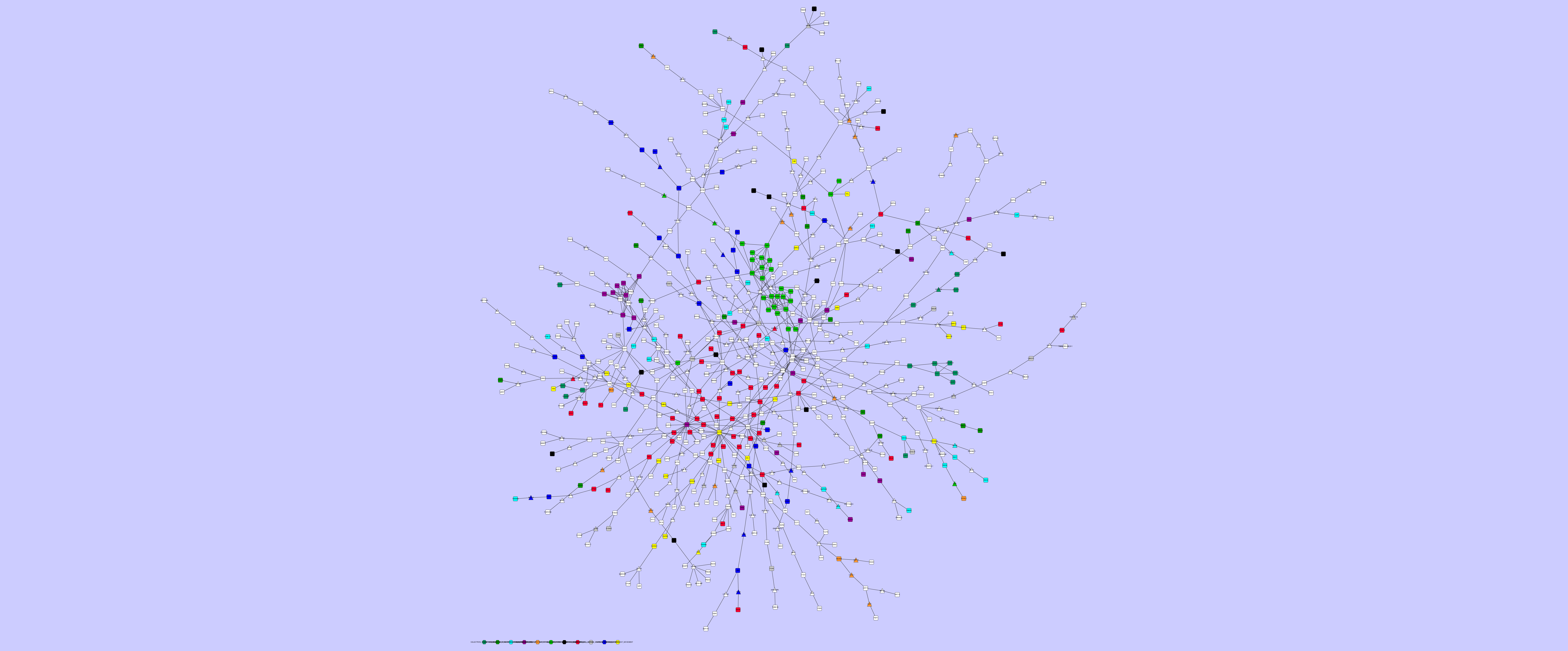

Supplement: Supplementary file 3 — S3: PPI network of eel gills [file mmc3.zip › S3.png]
